# Converting the “union curious”? Rights-based, pro-worker arguments and Republican support for expanding collective bargaining: The case of the Illinois Workers’ Rights Amendment

**DOI:** 10.1371/journal.pone.0335702

**Published:** 2026-02-06

**Authors:** Nicholas W. Waterbury, Magic M. Wade, Alan J. Simmons

**Affiliations:** University of Illinois at Springfield, Springfield, Illinois, United States of America; National Cheng Kung University, TAIWAN

## Abstract

In 2022 Illinois voters were faced with a ballot measure asking them whether they supported adding a Workers’ Rights Amendment (WRA) to the state constitution. Despite countervailing forces that might have made passage difficult, the amendment passed. We explore whether support for collective bargaining rights and union protections followed a predictably partisan pattern in Illinois, or whether support for the amendment was shaped by arguments, endorsements, or other voter demographics. Fielding a survey experiment with a representative sample of 1,000 Illinois voters, we find that Democrats were more likely to support the WRA in general, but that Republicans were more likely to support it following exposure to rights-based arguments emphasizing better pay, benefits, and conditions for workers. We also find that Democrats were more likely to support it following exposure to public sector union endorsements, but that private sector endorsements did not sway Republicans. More broadly, these findings suggest future opportunities to influence potentially skeptical audiences when it comes to ballot measures related to the labor movement.

## An auspicious victory

On November 9, 2022, Illinois voters approved Constitutional Amendment 1, the “Right to Collective Bargaining Measure.” Colloquially known as the Workers’ Rights Amendment (WRA), the measure amends the Illinois constitution to establish the right of employees to organize and bargain collectively through representatives of their choosing for the purpose of negotiating wages, hours, and working conditions, and to protect their economic welfare and safety at work. Moreover, it prohibits lawmakers from enacting legislation that infringes upon employees’ collective bargaining rights to prevent the implementation of “right-to-work” legislation in Illinois. The passage of the WRA, the first state law of its kind enacted through direct democracy, begs the question: what shapes public attitudes toward expanding labor protections?

To ascertain how individual and contextual factors may have influenced attitudes toward the ballot measure, we fielded a survey about the Illinois Workers’ Rights Amendment to likely voters that includes experimental treatments and arguments. Reinforcing established wisdom, Democrats were generally more supportive of the WRA. However, Republicans could be persuaded to support the WRA following exposure to rights-based arguments emphasizing the amendment as a vehicle for improving workers’ pay, benefits, and employment conditions. These findings illustrate opportunities for voter referenda campaigns focused on labor issues to broaden their base of support while highlighting the role of arguments in policy attitude formation, particularly among cross-pressured or ambivalent partisans.

Organized labor achieved a significant milestone with Illinois Amendment 1, which made Illinois the first US state to enshrine collective bargaining rights in its constitution. Substantive changes to the Illinois constitution must be proposed via a joint legislative resolution and approved by 3/5 of the members of both houses before being placed on the next general election ballot. Ballot measures must then be approved by over half of the Illinois electorate (as was the case with the WRA) or 60% of votes cast on the question. Since broad support from lawmakers and voters is now required to repeal the WRA, a renegade anti-union state legislature cannot enact right-to-work laws in Illinois. By contrast, 27 states presently have right-to-work policies, including nine constitutional measures. Bucking such trends, the WRA’s passage was a substantial policy achievement for the labor movement in Illinois.

Passage of the WRA was also an important political victory for Governor Pritzker and his allies after having failed to pass another high priority agenda item through a voter referendum in 2020: the Fair Tax Amendment (FTA). The FTA would have enabled a graduated income tax on the state’s highest earners and was endorsed by the same broad coalition of prominent Democrats, organized labor, and advocacy organizations as the WRA. Nonetheless, only 45% of voters supported the FTA, while 58% supported the WRA. Given that similar political coalitions mobilized for both measures, what explains Illinois voters’ attitudes toward the WRA? To better understand Illinois voter attitudes toward the WRA and how it passed, we fielded a survey experiment with a representative sample of likely Illinois voters before the 2022 midterms building on previous attempts to understand attitudes towards labor policies. The survey measured respondents’ support, opposition, or ambivalence toward the WRA alongside individual attributes such as partisanship, union relationship, education, and more. For our experiment, we exposed respondents to referenda language treatments focused on arguments for and against the WRA, along with interest group endorsements of the WRA.

We combine insights from labor studies and political science to highlight determinants of public attitudes toward expanding labor protections through direct democracy. We find that partisans had stronger attitudes toward the WRA than independents, with Democrats being supportive and Republicans being opposed. But while partisans were the most opinionated, they were also persuadable. Democrats were even more supportive when exposed to friendly WRA endorsements by public sector unions. Republican skepticism decreased when presented with right-based arguments that its passage would improve employees’ material conditions, building on works that looked at the impact of compelling and values-based arguments on voter decision making [1,2].

Republicans’ amenability to expand collective bargaining rights when presented with right-based arguments emphasizing better pay, benefits, and working conditions, coupled with the observation that the WRA outperformed all statewide Democratic candidates, signals a shift in public sentiment observed a mere decade earlier, during the Great Recession, when numerous Republican governors, including Bruce Rauner of Illinois, ascended on promises to weaken unions through the erection of draconian right-to-work laws. As well, support for organized labor plummeted to an all-time low during this era, but by 2022 had returned to near-historical highs [3].

Our analysis of what factors may have impacted voter attitudes toward the WRA suggests that policies strengthening unions have bipartisan appeal when framed in ways emphasizing collective bargaining as a right that, when exercised, improves workers’ material and employment conditions. In addition to mobilizing the labor movement’s loyal base of Democratic supporters, advocates seeking to expand union protections through statewide referenda may want to court persuadable and/or ambivalent partisans and independents in their outreach and messaging through right-based arguments focused on the economic benefits of such policies.

## Americans’ support for organized labor: Ambivalence as persuadability?

Decades of research show that union membership and higher union density improve employees’ wages and working conditions [4]. American workers employed outside of unionized industries also benefit from positive spillover effects of unionization like increased minimum wages [5]. Nonetheless, the American public holds a fickle attitude toward organized labor. For instance, Gallup reported that 71% of Americans approved of labor unions in 2022, a figure that had been steadily increasing from an all-time low of 48% in 2009 and remained at 70% in 2024, within the poll’s margin of error [6]. At the same time, 61% of Americans said unions helped rather than hurt the economy, and 77% said unions mostly help their members [7]. Still, 58% of non-unionized workers in 2022 said they were “not interested at all” in joining a union [7]. What explains Americans’ ambivalence toward labor unions?

Union decline [8] negative media portrayals [9,10], and class conflict [11] have been cited to explain the relative skepticism toward and/or unpopularity of labor unions. Additionally, partisanship, policy preferences, socialization, and ideology largely shape Americans’ attitudes toward organized labor and explain the large, enduring gap in support for unions among Democrats and Republicans [12,13]. Exemplary of this, unions enjoyed a 45-point advantage in Democratic (94%) over Republican approval (49%) in 2024. Unionized as well as swaths of non-unionized workers are loyal to the Democratic Party due to policy congruence rooted in the belief that unions are a vehicle for improving the pay and working conditions of workers [12,14].

Republicans, on the other hand, frequently associate unions with the Democratic Party, public employee unions, budget crises, higher taxes, labor bosses, and communism [15–18]. Nonetheless, Republican approval for unions increased a striking 23 points in 2024 (49%) over 2011, when it cratered at 26% [6]. Likewise, approval of unions among independents has hovered steadily around 65–69% since 2020. Notwithstanding lingering union skepticism and/or hostility espoused by roughly half of Republicans, there has been a relatively steady bipartisan resurgence of support for labor unions in the US since 2014 [6]. Such incongruence between recent and long-observed patterns of partisan polarization toward unions is puzzling, and potentially pivotal for expanding collective bargaining rights through direct democracy.

Relatedly, Donald Trump’s outreach to union voters during his 2024 presidential campaign received much media attention. Exit polls subsequently suggested Trump’s erosion of the union was relatively small, while labor strategists like Steve Rosenthal characterized Trump’s policy agenda as “anti-worker” [19]. Nonetheless, the Republican Party of Donald Trump in 2024 was arguably symbolically more labor-friendly than the Republican Party of a decade earlier, when red-state governors who gained notoriety for implementing right-to-work regimes in their states like Scott Walker (R-Wisconsin), Rick Snyder (R-Michigan) and John Kasich (R-Ohio) were touted as presidential contenders [20]. However, this does not mean a pro-labor paradigm shift marked by substantive support for expanding collective bargaining rights and union protections has overtaken the GOP. Rather, political expedience has long characterized the party’s relationship to organized labor.

Two studies spanning over five decades found that Republican state and congressional lawmakers representing labor strongholds pandered to unions by voting for Democratic-sponsored, union-endorsed labor-relations bills [21] or by “shielding” their labor allies from occupation-based collective bargaining reforms [22]. Republican outreach to unions and their supporters may be novel, but it is not unprecedented. Lyon [23] found that Republicans from union households and those working in traditional low-wage occupations (regardless of union status) were more supportive of unionizing for various occupations of workers than Republicans without such experiences. Moreover, Democrats’ and Republicans’ support for unions varied by worker occupation type, with low-wage Republicans being most supportive of unions representing retail and fast-food workers and Democrats being more supportive of unions representing teachers and manufacturing/factory workers than fast-food, retail, and police/fire fighters’ unions [23]. Following this, the strength of partisanship in determining attitudes toward a pro-labor policy like the Illinois Workers’ Rights Amendment might be weaker in Republicans who are unionized, working-class, and/or have a weaker partisan attachment.

Then, while discrete pockets of union-supporting Republican elites and rank-and-file voters have been identified by previous research and are expected to be more sympathetic to the WRA, it’s also noteworthy that a sharp bipartisan attitudinal shift in favor of organized labor appears to be underway. Recent survey research by Alquist et al. [24] suggests this attitudinal shift is characterized by increased support for organized labor, but also the replacement of opposition against unions with a more “ambivalent” posture characterized by more people recently saying they “don’t know” if they would vote to join a union to represent them rather than offering an outright “no” response. Alquist et al. argue that US workers are increasingly “union curious,” especially those under 30 [24]. This may be rooted in unawareness of partisan cues urging them to support/oppose unions, or lack of compelling cues to that effect. Interestingly, only 27% of survey respondents agreed that unions were controlled by Democrats, while 31% disagreed and 42% responded “didn’t know” [24]. The authors describe this as “concerning,” but ambivalence about whether unions are controlled by Democrats presents an opportunity for pro-labor legislation to reach the growing ranks of “union curious” Republicans and Independents/unaffiliated voters.

Considering this, we anticipate that Republicans with weaker partisan identification, low-wage/blue collar work experience, and/or those who reside in union households will exhibit higher than expected (based on partisanship) support for the WRA. Moreover, even Republicans who recognize the Democratic Party’s strong ties to organized labor and support for collective bargaining rights may be persuaded to support pro-labor policies if they are presented with a compelling, substantive rationale or an endorsement from a blue-collar/private sector labor union (as opposed to a white collar/public employee union). We expect such voters will become more supportive toward the WRA when presented with arguments that emphasize its economic benefits to the working class. This is based on political scientists’ understanding that voters rely upon mental shortcuts to evaluate policy proposals instead of systematic policy knowledge [25,26].

While partisanship and elite cues are among the key heuristics shown to structure vote choices on ballot measures [27–30], less conventional heuristics include interest group endorsements [31,32] and appeals to basic values [33].

Relatedly, Stanley et al. [34] found that after being exposed to compelling arguments supporting their opponents’ policy positions, hostility toward the outgroup was diminished. Extending this, Tappin [2] found that the positive effect of “persuasive messaging” about various policies on respondents’ support of them was undiminished by countervailing cues from respondents’ “in-party” leaders. Similarly, Tappin et al. [35] found that voters independently “integrated” substantive arguments in favor of policies with information about their party leader’s positions on such policies, with both pieces of information influencing partisans’ policy attitudes.

If partisanship is a key heuristic [25], and partisan world views are increasingly shaping attitudes and opinions more than other identities [36–38], where does that leave independent voters? Especially “true independents” that do not have a partisan lean [39,40]? Absent strong party loyalty, research finds independents are more likely than partisans to be swayed by arguments made about policies [41–43]. In particular, independents are more likely to be swayed by interest groups, such as unions used in this study [44,45]. Further, independents are less impacted by messaging that focuses on negatives compared to positives for policies or candidates [46], though some work finds no difference across type of messaging for independents [47]. Lastly, some of this work finds the impact to independents to be limited [44] or no different compared to partisans [47].

Then, endorsements of the Workers’ Rights Amendment from specific types of unions—those representing blue-collar/private sector versus white-collar/public sector workers are expected to differently appeal to partisans. A robust strand of political science research links the political clout of public employee unions to state-level fiscal and policy outcomes [17,18,48,49]. Conservative dogma bolstered by such research dictates that weakening public sector unions is necessary to reduce government expenditures and increase voter control over policies. Resultantly, the Republican Party’s concerted effort to restrict collective bargaining rights surrounding the Great Recession (2008−2012) focused primarily on public sector unions, especially those representing K-12 teachers [22] Media and elite discourse portrayed public employees as overpaid and underworked, and their unions as blameworthy for the economic crisis [16]. Ideological opposition to collective bargaining in the government sector was thus a key driver of anti-union sentiment among Republicans during this time [50]. For this reason, Republicans are expected to possess ideological reasons to oppose the WRA when they are informed it is endorsed by government employee unions as opposed to trade unions, while Democrats support for the WRA is expected remain high regardless of which unions are said to endorse the amendment.

Following this, in addition to partisanship, heuristics available for the WRA might include endorsements from labor unions and arguments supporting the measure that frame it using right-based language that appeal to material values. When presented with these, partisanship might be diminished as a heuristic, leading Republicans to be more supportive of the measure.

In sum, the sharp rise in Republican approval of unions in tandem with Democrats’ increasing support is a noteworthy development in American Politics with potential implications for the ability of unions to mobilize voters to support expanding collective bargaining rights through direct democracy. We explore this possibility by examining the malleability of Republican and Independent voter support for expanding labor protections in the context of the Illinois Workers’ Rights Amendment.

## Partisanship and the WRA

Party leaders and state lawmakers occupied partisan, contrary positions towards Illinois Amendment 1 during legislative debate and the ballot measure campaign. First, Democrats in the Illinois General Assembly were overwhelmingly supportive of the joint resolution that placed the Right to Collective Bargaining Measure on the 2022 ballot, which 73 Democrats co-sponsored compared to only four Republicans. Governor Pritzker also campaigned explicitly in support of the WRA, proclaiming, “We need to make union organizing a constitutional right and stop Republican efforts to eliminate collective bargaining” [51]. Then, Illinois trade unions were among the largest financial contributors to the “Vote Yes For Workers’ Rights Campaign,” which outspent the “Vote No” campaign by a margin of 5:1 [52]. Donors contributing one million or more dollars to the “Vote Yes” campaign included the Midwest Region Laborers, Chicago & General Laborers, Illinois Pipe Trades Association, International Union of Operating Engineers, and the United Brotherhood of Carpenters [53].

By contrast, vocal opponents of the WRA included Republican Gubernatorial candidate and State Senator Darren Bailey, who said of the amendment, “It’s a special interest takeover that has nothing to do with workers’ rights.” Likewise, at least five newspaper editorial boards urged voters to reject Amendment 1, arguing it would negatively impact the state’s business [51,54]. Finally, owner of Uline and Schlitz Brewing, the billionaire Richard Uihlein, a frequent supporter of conservative candidates and policies, emerged as the primary campaign contributor against the ballot measure [52].

Considering positions espoused by elites, interest groups, and media toward the WRA, plus context clues in the ballot measure itself, Illinois voters may have perceived the ballot measure as Democratic-coded and voted accordingly. Our partisanship hypothesis presumes that Democrats from all economic strata will be predictably positioned (i.e., supportive) of expanding labor protections through the amendment. We then expect partisanship to drive more Republicans to oppose the WRA than Democrats but hypothesize this will be mitigated among Republicans with weaker partisan identification, low-wage/blue collar work experience, and/or those who reside in union households.

Next, our persuadability hypothesis anticipates that independents, weak Republicans, and union and/or working-class Republicans will become more supportive toward the WRA when presented with arguments that emphasize its economic benefits to the working class, while Democrats are expected to have strong support regardless of exposure to supportive arguments.

We use two union endorsement treatments to test whether partisans experience more cross-pressure about expanding CB protections when they learn the WRA is endorsed by (i.e., perceived to benefit) private sector/blue collar worker unions or public employee/white collar unions. In actuality, all unions listed in either treatment supported the WRA campaign through endorsements and financing. Given the Democratic Party’s strong association with public employee unions and Republicans’ ideological opposition toward public sector collective bargaining, we expect negative partisanship to be strongest when Republicans are informed that the WRA is endorsed by AFSCME, the Chicago Teachers Union, and Illinois Federation of Teachers. Then, we anticipate Republican support will either be unaffected or positively affected when the WRA is depicted as endorsed by the United Brotherhood of Carpenters and Joiners of America, International Union of Operating Engineers, and UA of Journeymen & Apprentices of the Plumbing & Pipefitting Industry. Democrats are expected to support the WRA under both endorsement treatments, although we anticipate slightly lower support for the amendment when it is associated with trade unions, since this might send a weaker partisan signal to Democrats about where the party stands on the WRA than a teacher union endorsement.

Building on the above thinking, we propose the following hypotheses:

Partisan hypotheses

Support/opposition to the WRA will be structured by partisanship, with Democrats more likely to support and Republicans less likely to support.

Persuadability hypotheses

Independents will be persuadable when presented with arguments for or against the WRA. Republicans and Democrats are less likely to be persuaded with arguments for or against the WRA.

Endorsement hypothesis

Democrats will be more likely to support the amendment when it is presented as supported by public sector groups. Republicans will be more likely to support the amendment when it is presented as supported by private sector trade unions.

## Research design and data

To test our hypotheses and better understand Illinois voter attitudes toward the Workers’ Rights Amendment we fielded a survey with likely Illinois voters before the 2022 midterm elections. The survey measured individual attributes such as partisanship, union membership, education, etc. and exposed voters to referenda language treatments including arguments for/against and endorsements alongside measures of support, opposition, and ambivalence toward the WRA. The research design and the contents of the survey were approved by The University of Illinois, Springfield Institutional Review Board in Fall 2022. Written informed consent was obtained through a waiver electronically signed by respondents prior to participation. The sample was made up of 1,000 respondents and was representative of the Illinois voting population based on previous exit polls in Illinois [55]. The sample was representative with respect to race, education, income, religion, partisanship, gender, and geographic location. For certain characteristics where our sample was not representative (e.g., union membership), we weight the survey responses to be consistent with statewide demographics (Our online panel was provided through Marketing Systems Group and the survey respondents completed the survey through the online Qualtrics Research Suite. The survey was fielded from October 17, 2022 – October 25, 2022. Successful respondents passed attention checks within the survey in keeping with best practices [56–58]. Table 1 compares weighted sample characteristics to population benchmarks across key demographic, socioeconomic, religious, and political variables. Overall, the weighted sample closely approximates the population distributions with modest deviations for education, religious affiliation, and ideological self-placement. Respondents were required to answer every question in order to complete the survey. As a result, no respondents are excluded from any part of our analyses due to missing data nor was any form of multiple imputation required.

**Table 1:** Comparison of weighted sample with population benchmarks.

Variable	Weighted Sample	Population Benchmarks
**Gender**		
Male	46	47
Female	54	53
**Age**		
18-29	11.2	12
30-44	25	24
45-64	38.8	37
65+	25	27
**Education**		
High School or Less	21.6	24
Some College	33.9	33
Bachelor’s Degree	31.3	26
Postgraduate Degree	13.2	16
**Union Membership**		
Union household	21.86	22
Nonunion household	78.14	78
**Religion**		
Catholic	33.4	29
Other Christian	32.3	38
Jewish	3.6	3
Muslim	2.1	1
Not Listed Religion	16.5	8
**Income**		
Under $25,000	14.1	15
$25-49,000	24.3	23
$50-74,000	22.3	21
$75-99,000	14.1	16
Over $100,000	25.2	25
**Political Party**		
Democrat	54	54
Republican	41	41
Independent	5	5
**Ideology**		
Very Liberal	9.6	14
Somewhat Liberal	25.4	20
Moderate	31.9	34
Somewhat Conservative	26.4	18
Very Conservative	6.7	14

*Note:* Population benchmarks courtesy of exit polls collected by the *New York Times* [55]. Sample weighted on union household membership.

Surveying Illinois on this issue is useful for several reasons. First, the only other state to pass a similar ballot measure to enshrine collective bargaining rights in its constitution was Hawaii, in 1968. Missouri labor advocates attempted, but failed, in 2022 to garner enough signatures to add a similar initiative to the state’s 2022 ballot. Illinois therefore presents the best, most recent opportunity to understand attitudes towards such an initiative due to the lack of historical data availability in other cases and the opportunity for the research team to collect data with the treatment effects proposed here. Second, the findings from our study of Illinois can be useful for understanding attitudes towards such initiatives more widely. Illinois has a diverse political landscape across race, ethnicity, geography, ideology, education, ideology, and income. In fact, some research suggests that Illinois is the state which most accurately represents America when it comes to key demographics [59,60]. Further, areas of Illinois have been utilized by US Presidential campaigns for decades as a means for understanding political attitudes of Americans more widely [61–63].

Relatedly, while conventionally viewed as a stronghold for the Democratic Party, a closer look suggests that Illinois is more of a political battleground state than meets the eye. For example, 54% of the state belong to the Democratic Party or “lean” towards them [55]. As well, in 2022 Illinois has a partisan voting index of +7, placing it in the lower half of states that Democratic-positive scores [64]. Further, while Democrats swept statewide offices in 2018 and 2022, in 2020 the Democrat-backed “Fair Tax” amendment to the Illinois constitution that would have allowed for a graduated income tax was rejected by a majority of voters. Additionally, while Democrats won a larger percent of the statewide vote for the Illinois Senate in 2022, Republicans won a slight majority of the statewide House vote, though Democrats won the most seats in both. This all suggests that while perhaps being more favorable towards Democrats, Illinois voters have a more complex relationship with the party and the policies they support like the WRA.

### Dependent variable

We focus on a single dependent variable in this analysis: **Support for the WRA. Support for the WRA** is a binary variable, coded as 1 if the respondent indicated they supported the Workers’ Rights Amendment at the conclusion of our survey and 0 if they opposed the amendment or were undecided. In total, 47.7 percent of the respondents indicated their support for the WRA. The exact question wording asked respondents:

“How do you plan to vote on the Right to Collective Bargaining Amendment that will be on the ballot November 8?”

Astute observers will notice the discrepancy between the level of support for the WRA in this sample compared to the actual results in the 2022 statewide election. We expect this is because of the number of late deciding voters---in our sample nearly one quarter of voters told us (post-treatment) they were undecided on the WRA---and the high abstention rate in the actual statewide vote on the measure.

### Variables of interest

Our hypotheses require multiple variables of interest. Beginning with partisan affiliations: **Democrat** is a binary variable coded as 1 if a respondent self-identified as a member of the Democratic Party, including those who identify as independents but “lean” Democrat; **Republican** is a binary variable coded as 1 if a respondent identified as a member of the Republican Party, including those who identify as independents but “lean” Republican. In our sample, 54 percent of respondents were Democrats and 41 percent were Republicans. A distinct measure, **Independent** is a binary variable coded as 1 if respondents did not identify with either of the two major parties even after they were “pushed” to declare the party they “leaned” closer to. Independents here then are “true” independents and not undercover partisans [39,40]. Five (5) percent of respondents identified as true Independents, comparable to previous work in this area.

In addition to these partisan indicators, we randomly exposed a subset of respondents to a series of vignettes to gauge the malleability of the likely voters in our survey. Each respondent had an equal chance of being exposed to each treatment. All respondents received an explanation of the WRA derived from the language used on the ballot:

The Right to Collective Bargaining Amendment (known as “Amendment 1”) that will be on the ballot November 8 would change Article I of the Illinois Constitution to add the following language: Employees shall have the fundamental right to organize and to bargain collectively through representatives of their own choosing for the purpose of negotiating wages, hours, and working conditions, and to protect their economic welfare and safety at work. No law shall be passed that interferes with, negates, or diminishes the right of employees to organize and bargain collectively over their wages, hours, and other terms and conditions of employment and workplace safety, including any law or ordinance that prohibits the execution or application of agreements between employers and labor organizations that represent employees requiring membership in an organization as a condition of employment.

An initial group only received that descriptive information provided above and no additional information.

The first treatment group, in our analysis identified by the indicator variable **Pro-WRA Arguments**, received a vignette that included the explanation of the WRA and contained the additional sentence:

Supporters of the amendment argue the “amendment will update the Illinois constitution to guarantee every Illinoisan has the right to join together with other workers to negotiate for better pay, improved benefits, and safe working conditions.”

A second treatment group received an additional sentence with an opposition argument:

Opponents of the amendment argue “approving Amendment 1 would not protect all Illinois workers, only the special interests who want to put themselves above state law. What it would do is raise taxes on every Illinoisan to pay for excessive benefits for those select few.”

A third group of respondents received a second treatment focused on endorsements. This group, indicated by the binary variable **Public Sector Endorsement**, received the explanation and a list of public sector unions or organizations that supported the WRA along with a list of business lists that opposed the WRA:

The Amendment is supported by the following groups:
  AFSCME Council 31
  Chicago Teachers Union
  Illinois Federation of Teachers
The Amendment is opposed by the following groups:
  Associated Builders and Contractors
  Illinois Chamber of Commerce
  Illinois Manufacturers’ Association

Finally, a final group, indicated by the binary variable **Private Sector Endorsement**, received the explanation and a list of private sector unions or groups that supported the WRA along with a list of business interests that opposed the amendment:

The Amendment is supported by the following groups:
  United Brotherhood of Carpenters and Joiners of America
  International Union of Operating Engineers
  UA of Journeymen and Apprentices of the Plumbing and Pipefitting Industry
The Amendment is opposed by the following groups:
  Associated Builders and Contractors
  Illinois Chamber of Commerce
  Illinois Manufacturers’ Association

The distribution of treatment assignment is presented in Table 2. The distribution suggests that our randomized assignment of treatment groups was successful and allows us to operate under many of the favorable assumptions that exist in that context [65]. Further, an *a priori* power analysis revealed that in a comparative analysis of the five treatment groups with one another, at conventional alpha (0.05) and power (0.80) levels, to capture a medium effect size would require a sample of approximately 500 respondents in total or 100 in each treatment group. The a priori power analysis rests on several explicit assumptions. First, we assume a medium-sized average treatment effect, operationalized as Cohen’s *f* = 0.25, consistent with conventional benchmarks in the social sciences. Second, we assume a five-group experimental design analyzed using a one-way ANOVA (or equivalently, an F-test from a linear regression framework), with approximately equal group sizes. Third, the analysis assumes a two-sided significance level of α = 0.05 and a desired statistical power of 0.80. Fourth, observations are assumed to be independent, and outcome variance is assumed to be approximately equal across treatment groups. Fifth, the power calculation pertains to detecting average treatment effects and does not imply guaranteed covariate balance across treatment and control groups in finite samples. Finally, the analysis does not assume sufficient power to detect interaction effects or heterogeneous treatment effects across multiple subgroups, which typically require substantially larger sample sizes.

**Table 2:** Treatment assignment groups.

Vignette(s) Received	Total Number of Respondents	Democrats	Republicans	Independents/Third Party
*Informative Vignette Only (Control Group)*	**197**	98	92	7
*Information and Pro-WRA Arguments*	**203**	112	79	12
*Information and Anti-WRA Arguments*	**202**	104	84	14
*Information and Public Sector Endorsements*	**202**	110	85	7
*Information and Private Sector Endorsement*	**196**	116	92	10

### Covariates

We also account for a series of pre-treatment characteristics of a respondent that could be predictive of attitudes on the WRA and variables of interest like partisanship. The binary variable **Female** captures the respondent’s self-identified status as a female. The binary variable **White** records if the respondent self-identified as non-Hispanic White. The ordinal **Income** is a scaled measure of the respondent’s self-identified income. The ordinal **Age** is a scaled measure of a respondent’s self-identified age. The binary variable **Unemployed** captures if a respondent was out-of-work or unable to work (retired individuals included). The binary variable **Union Household** measures if the respondent self-identified as a member of a union or if they identified any member of their household as a member of a union. The binary **Working Class** variable is coded according to the measure as defined by Carnes and Lupu [66]. Respondents are considered working class if their self-identified employment is in manual labor, the service industry jobs, clerical work, or a union job. Finally, **Ideology** is a seven-point scale of a respondent’s political ideology ranging from ‘Extremely Liberal’ to ‘Extremely Conservative.’ More detailed descriptions along with the descriptive statistics for all variables are included in the S1 Appendix.

## Modeling strategy

A preliminary look at our initial results reveals that 47.7% of respondents support the WRA. Divided by partisan identification, we observed that Democrats are both far the most supportive of the proposal. A supermajority, 64.6% of Democrats in our sample support the WRA. This is much greater than the 27.3% of Republicans and 32% of Independents/Third-Party respondents that support the WRA. In addition to depicting these descriptives Table 3 also highlights a comparison between a partisan group and all other respondents through a difference in means test (the full report of the partisan bivariate tests is available in Table S5 of the S1 Appendix). This test reveals that Democrats are more likely at a statistically significant rate to support the WRA than all other respondents in the sample. In contrast, Republicans are significantly less likely to support the WRA than all other respondents. The same is true for Independents/Third-Party respondents who are likewise significantly less likely to support the WRA than other respondents.

**Table 3:** Aggregate support for WRA By partisanship.

	All Respondents	Democrats	Republicans	Independents/Third Party
*WRA Support*	**47.7%**	64.6%*	27.3%*	32.0%*

Note: * = statistically significant result at the.05 alpha level. All results reflective of a difference in means test between the partisan group and all other respondents (e.g., Republicans vs. Democrats and Independents).

An initial examination of treatment effects yields less conclusive results, at least in the aggregate. As demonstrated in Table 4, respondents in the control condition were slightly more likely than not to support the WRA. This was on par with respondents that received the pro WRA arguments and more than the respondents that received all other treatments. We conducted similar bivariate analysis to test for differences between the support of respondents in the individual treatment conditions and respondents in the control group (the full report of the bivariate tests for treatment effects is available in Table S6 of the S1 Appendix). There were no statistically significant differences between any of the individual treatment groups and the control group at the aggregate level.

**Table 4:** Support for WRA by treatment condition and partisan identification.

Vignette Received	Aggregate WRA Support	Democrat WRA Support	Republican WRA Support	Independent WRA Support
*Informative Vignette Only (Control Group)*	50.7%	69.4%	31.5%	53.6%
*Information and Pro-WRA Arguments*	50.7%	65.2%	35.4%	36.5%
*Information and Anti-WRA Arguments*	42.6%	56.7%	26.2%	35.0%
*Information and Public Sector Endorsements*	47.0%	67.3%	23.5%	43.2%
*Information and Private Sector Endorsement*	47.4%	64.7%	18.6%	31.4%

Table 4 also displays support for the WRA by treatment condition and partisan identification. In general, Democrats were more likely to support the WRA, Republicans were less likely, and Independents were somewhere in the middle. The effects of the treatment conditions vary within partisan groups. Democrats were particularly affected by anti-WRA arguments, while Republicans were influenced by pro-WRA arguments.

Still, as we have stated, there are theoretical expectations for why certain treatment conditions will not be received uniformly by all respondents. Further, this combined with the limited number of respondents in certain partisan/treatment groups suggests randomization alone is not likely to produce a dataset with covariates balanced by treatment condition. For this reason, while these initial results produce some interesting findings more detailed tests of our hypotheses are required. Best practices suggest that when imbalance and statistical power are concerns in an experimental design with multiple treatments of interest, to draw comparisons between a theoretically derived treatment group and all other respondents that did not receive the treatment condition of interest [67]. To account for the effects of other treatment conditions not of interest in a particular test, all treatment conditions are included in the relevant model as control variables. We therefore draw comparisons in the analyses that follow between the treatment effects derived in our hypotheses and all other respondents that did not receive a given treatment.

Because all our dependent variables are dichotomous, we fit a series of linear probability models with robust standard errors to account for heteroskedasticity. We make use of linear probability models for ease of interpretation. Because there are many well-known shortcomings of linear probability models including heteroskedasticity and unrealistic behavior in the tails of the distribution, we also fit logistic regression models for all of the analysis shown here. Those results, which are substantively similar, are presented in the Tables S2–S4 of the S1 Appendix. We fit three models to test the effect of identifying as Republican, Democrat, and Independent, respectively on support for the WRA. In order to perform the most straightforward test of the individual effect of party membership – and to avoid the complications of potentially overfit models [68] – we fit three separate models for each partisan indicator with control variables. As a robustness check, we also fit a model with both **Democrat** and **Republican** indicator variables included. The results suggest Democratic Party identification is a strong factor in WRA support. The effect is so pronounced that it minimizes the effect of Republican identification in the model. Importantly, we include indicators of any treatment conditions received. Thus, we can fit this model on the entire sample of respondents to examine the effect of partisanship even when accounting for other possible treatment effects.

## Results

Presented in Table 5 is a full report of the effects of different partisan affiliations on support for the WRA. The results are consistent with the Partisan Hypothesis. The table reports estimates derived from a linear model with robust standard errors in parenthesis. Coefficients with a star are statistically significant at the 0.05 alpha-level when a one-sided t-test is performed.

**Table 5:** The effect of partisanship on WRA support.

	*Dependent variable:*			
	*Support for WRA*			
	(1)	(2)	(3)	(4)
Democrat	0.204^*^			0.258^*^
	(0.040)			(0.070)
Republican		−0.153^*^		0.067
		(0.041)		(0.071)
Independent			−0.178^*^	
			(0.068)	
Union Household	0.134^*^	0.133^*^	0.130^*^	0.134^*^
	(0.038)	(0.038)	(0.038)	(0.038)
Pro-WRA Argument	−0.015	−0.017	−0.004	−0.013
	(0.046)	(0.047)	(0.047)	(0.046)
Anti-WRA Argument	−0.100^*^	−0.106^*^	−0.098^*^	−0.098^*^
	(0.046)	(0.046)	(0.046)	(0.046)
Public Sector Endorsement	−0.043	−0.042	−0.037	−0.043
	(0.045)	(0.046)	(0.046)	(0.045)
Private Sector Endorsement	−0.067	−0.068	−0.058	−0.065
	(0.046)	(0.047)	(0.047)	(0.047)
Working Class	−0.025	−0.022	−0.017	−0.025
	(0.033)	(0.033)	(0.033)	(0.033)
Ideology	−0.077^*^	−0.086^*^	−0.113^*^	−0.079^*^
	(0.011)	(0.011)	(0.008)	(0.011)
Income	−0.012	−0.012	−0.013	−0.012
	(0.010)	(0.010)	(0.010)	(0.010)
Age	−0.019	−0.015	−0.018	−0.020
	(0.017)	(0.017)	(0.017)	(0.017)
Female	−0.040	−0.038	−0.033	−0.039
	(0.029)	(0.029)	(0.029)	(0.029)
White	0.019	0.006	−0.021	0.017
	(0.037)	(0.038)	(0.037)	(0.037)
Unemployed	−0.016	−0.019	−0.029	−0.017
	(0.044)	(0.045)	(0.045)	(0.044)
Constant	0.801^*^	1.007^*^	1.083^*^	0.757^*^
	(0.092)	(0.076)	(0.075)	(0.104)
Observations	1,000	1,000	1,000	1,000

Note: All linear probability models.

* p < 0.05.

The results largely align with what one would expect from the descriptive tables above. The **Independent** and **Republican** coefficients are negative and statistically significant indicating respondents with those affiliations were less likely to support the amendment. The **Democrat** coefficient is positive and significant, indicating they were more likely to support the amendment. Each coefficient represents the change in the predicted probability of support for the WRA associated with a one-unit increase in the predictor variable of interest holding all else constant. This means that a Republican respondent has a probability 0.154 *less* than other respondents of supporting the WRA just by being a Republican. An independent respondent is similarly 17.8 percent *less* likely to support the WRA than other respondents, while a Democratic respondent is 20.4 percent *more* likely to support the WRA than other respondents. In addition, we conducted several robustness checks using alternative baseline comparisons (e.g., Democrats vs. Republicans, Democrats vs. Independents, and Democrats vs. all others). These alternative specifications yield substantively similar results: Democrats are more supportive of the WRA than all other groups, Republicans are less supportive of the WRA than Democrats and all others, Independents are less supportive of the WRA than Democrats and all others; there is no significant difference between the support levels of Independents and Republicans. Full reports of these estimates are available in Tables S7–S9 of the S1 Appendix.

To test our Persuadability Hypothesis we replicated the models of **Support** with interactions between our argument treatment variables and partisan affiliation. While a straightforward model of the effect of the argument treatments on support for the WRA are inconclusive (see Model 4 in Table S3 of the S1 Appendix), we observe a *conditional* effect of the treatments on partisanship. As depicted in Table 6, Republican respondents that received arguments encouraging WRA support were significantly more likely to support the amendment at the end of our survey. Republicans that received the arguments supported the WRA were had an increased probability of WRA support of 0.136. This is in contrast to Democrats and Independents who received the same arguments but displayed no significant effect on their probabilities of support as a result.

**Table 6:** The effect of persuasion and partisanship on WRA support.

	*Dependent variable:*		
	Support for WRA		
	(1)	(2)	(3)
Pro-WRA Argument * Democrat	−0.085		
	(0.073)		
Pro-WRA Argument * Republican		0.141^*^	
		(0.075)	
Pro-WRA Argument * Independent			−0.249
			(0.169)
Pro-WRA Argument	0.026	−0.081	0.017
	(0.061)	(0.056)	(0.050)
Democrat	0.384^*^		
	(0.034)		
Republican		−0.369^*^	
		(0.035)	
Independent			−0.121
			(0.082)
Anti-WRA Argument	−0.092^*^	−0.104^*^	−0.085*
	(0.046)	(0.047)	(0.050)
Public Sector Endorsement	−0.042	−0.041	−0.023
	(0.046)	(0.047)	(0.049)
Private Sector Endorsement	−0.056	−0.061	−0.021
	(0.047)	(0.047)	(0.050)
Union Household	0.143*	0.141*	0.136*
	(0.041)	(0.041)	(0.043
Working Class	−0.049	−0.050	−0.058*
	(0.032)	(0.033)	(0.034)
Income	−0.015	−0.015	−0.018
	(0.010)	(0.011)	(0.011)
Age	−0.037^*^	−0.031^*^	−0.050^*^
	(0.017)	(0.017)	(0.018)
Female	−0.049	−0.049	−0.037
	(0.030)	(0.030)	(0.032)
White	0.028	0.014	−0.090^*^
	(0.037)	(0.038)	(0.039)
Unemployed	−0.009	−0.011	−0.031
	(0.043)	(0.044)	(0.047)
Constant	0.464^*^	0.821^*^	0.793^*^
	(0.076)	(0.074)	(0.077)
Observations	1,000	1,000	1,000

Note: all linear probability models.

One-sided test; ^*^p < 0.05.

Finally, to test our Endorsement Hypothesis we replicated the models of **Support** with interactions between our endorsement treatment variables and partisan affiliation. Table 7 reveals that, again, the conditional effects tell an interesting story. This time, however, the significant results involved Democrats. Democrats that received a signal of support from their traditional allies in the public sector were 11.3 percent more likely to support the WRA than other respondents. Non-Democrats were not influenced by the Public Sector Endorsement. Republican respondents did not respond to messages from their allies through the **Private Sector Endorsement** treatment.

**Table 7:** The effect of endorsements and partisanship on WRA support.

	*Dependent variable:*	
	Support for WRA	
	(1)	(2)
Republican * Private Sector Endorsement	−0.095	
	(0.072)	
Democrat * Public Sector Endorsement		0.113*
		(0.070)
Republican	−0.137^*^	
	(0.043)	
Democrat		0.181^*^
		(0.043)
Private Sector Endorsement	−0.032	−0.064
	(0.056)	(0.046)
Union Household	0.133^*^	0.136^*^
	(0.038)	(0.038)
Pro-WRA Argument	−0.015	−0.014
	(0.047)	(0.046)
Anti-WRA Argument	−0.105^*^	−0.100^*^
	(0.046)	(0.046)
Public Sector Endorsement	−0.041	−0.103^*^
	(0.046)	(0.057)
Working Class	−0.023	−0.028
	(0.033)	(0.033)
Ideology	−0.085^*^	−0.077^*^
	(0.011)	(0.011)
Income	−0.012	−0.012
	(0.010)	(0.010)
Age	−0.014	−0.020
	(0.017)	(0.017)
Female	−0.039	−0.042
	(0.029)	(0.029)
White	0.008	0.018
	(0.038)	(0.037)
Unemployed	−0.017	−0.019
	(0.045)	(0.045)
Constant	0.995^*^	0.820^*^
	(0.077)	(0.092)
Observations	1,000	1,000

Note: all linear probability models.

One-sided test; ^*^p < 0.05.

Still these results warrant discussion within the context of the aggregate effects in Tables 3 and 4. We observe that aggregate support in both the public and private sector endorsement treatments is lower than in the control condition and the pro-WRA argument condition. While the size of this difference is modest, it is notable given that these treatments were designed to provide positive cues in favor of WRA. One possible explanation is that the endorsements had minimal persuasive impact on their own, while simultaneously provoking backlash or skepticism among certain respondents, thereby reducing average support. This seems more likely than the alternative explanation that these endorsement cues produced differential effects across partisan groups leading to a net reduction in aggregate support because no partisan group experiences a significant increase in support following endorsements by their most likely allies. Although our current design does not allow us to fully explain this result, we highlight this as a potentially important asymmetry in how elite endorsements are processed. Understanding whether negative reactions systematically outweigh positive ones in response to policy endorsements is a fruitful direction for future research.

## Discussion

The findings of this study underscore the multifaceted dynamics at play in the passage of the Workers’ Rights Amendment (WRA) in Illinois and shed light on effective strategies for promoting pro-worker policies in diverse political landscapes. The successful advocacy for the WRA, predominantly driven by organized labor and Illinois Democrats, highlights the intertwined relationship between partisan alignment and support for labor rights initiatives. Moreover, the role of endorsements from public sector unions in bolstering Democratic support for the WRA underscores the importance of strategic alliances and messaging in shaping voter attitudes. Furthermore, our findings demonstrate how right-based appeals to improve pay, benefits, and working conditions can broaden support for labor protections, particularly among Republicans and independents who increasingly support unions in national surveys [7]. The remarkable performance of Illinois Amendment 1, surpassing electoral outcomes for Democratic candidates statewide and performing particularly better in the traditionally Republican-leaning region of the 95 Illinois counties outside of the Chicago area, suggests a potential power of pro-worker policies to appeal to a wider set of voters than partnership alone may allow for [69]. This phenomenon represents a noteworthy development in the landscape of labor advocacy. By emphasizing tangible benefits such as wage increases, proponents of workers’ rights may be able to transcend the strong influence of partisanship and garner wider support for their initiatives.

In total, our findings suggest that Democratic and pro-labor advocates may want to focus their attention more on arguments around the economic benefits of their policies when making appeals to voters who may be from more skeptical groups. This may be especially true as Republican politicians, voters, and conservative think tanks in Illinois remain mostly hostile toward collective bargaining rights, teachers unions, and the Democratic Party’s labor alliances [70,71]. This hostility from Republican elites may face challenges considering the widening base of Republican support among a non-college educated voters [72] coupled with increasing support for (or at least ambivalence toward) unions among Republicans and younger workers [7,24].

The US labor movement has been attracting more younger, college educated, and white-collar workers [73] while a “diploma divide” has emerged as one of the best predictors of partisanship and vote choice, especially among white voters [74]. Zacher [75] argues that the political realignment of Americans based on educational attainment and geographical sorting threatens the ability of the Democratic Party to advance an economically redistributive policy agenda. As the base of the Democratic Party has become more urban-dwelling, college-educated, and high-earning, its appetite for taxing those at the highest income distribution has waned. As evidence of this, Zacher observes that during the Obama years, Democrats promised not to tax families earning less than $250,000 annually; by 2020 Biden had raised this threshold to families earning less than $400,000. Congressional Democrats have also blocked efforts to reform tax deductions for mortgage interest and 529 college savings accounts, although these disproportionately benefit wealthier Americans [74]. This paradox applied to Illinois may help explain why the Illinois Fair Tax Amendment lost, but the WRA won. Although proponents of the Fair Tax argued that 97% of Illinoisans would see no tax increase (or a decrease) if constitutional amendment passed, opponents of the Fair Tax portrayed it as a tax on “working families” in their messaging because individuals and households earning over $250,000 would have been subject to a tax increase under new marginal rates [11].

Compared to redistributive policies like graduated income tax rates, “Affluent Democrats” identify with the working class and support collective bargaining rights on principle. They also support, as Zacher (2024) acknowledges, expanding the public sector and the welfare state, unlike Republicans who still claim they want to cut taxes and shrink government. With educational attainment, income, or one’s relationship to the means of production no longer satisfactory proxies for social class, the target audience for “pro-worker” legislation is unclear. Can this ambiguity be exploited by labor activists seeking to broaden their appeal? While the Democratic Party may find intraparty divisions an impediment to a redistributive agenda, we argue they should seek to shore up their base of Democratic support while finding common ground with working class Republicans by emphasizing the benefits of union representation for American workers.

Notably, union endorsements from public sector/white-collar or private sector/blue-collar unions did not demobilize support for the WRA among those already expected to be skeptical (i.e., Republicans and Independents). This was surprising considering the Republican Party’s strong stance against teachers and government employee unions. Is disdain for public sector unions no longer a unifying issue for Republicans? Relatedly, President Trump’s symbolic outreach to unionized workers during his 2024 campaign may increase Republican ambivalence toward organized labor despite decades of media coverage depicting unions and unionized workers as members of a “new undeserving rich” who materially benefit from union protections at the expense of non-unionized workers have been a mainstay of media coverage for decades [9,76]. Since exposure to negative portrayals of unions have been demonstrated to increase perceived differences between unionized and non-unionized workers and to demobilize non-unionized workers’ support for pro-labor policies [11], it is crucial for labor advocates to seize upon this moment.

Since Democrats already overwhelmingly support unions, advocates for strong union protections need to persuade ambivalent Republicans and independents to join their cause. Our research suggests that right-based arguments that appeal to values for better pay, benefits, and conditions for workers may have the potential to persuade the “union curious” to vote pro-labor. Dismantling longstanding partisan polarization in union support would have consequences for the future of the labor movement, since the large and enduring partisan gap in support for unions is an impediment to expanding collective bargaining rights through direct democracy. Although Republicans remain less supportive of unions generally and the WRA than Democrats, our research builds upon prior studies [24] about the growing ranks of “union curious” and recent polling data [6] to suggest that ambivalence among Republican and independents may be replacing outright union hostility, especially among the working-class.

Finally, our findings connect to other recent policy outcomes suggest that pro-worker referenda can succeed in non-Democratic strongholds by appealing to voters’ support for improving the material conditions of the working class. For example, majorities of voters in Florida in 2020 and Nebraska in 2022 approved statewide minimum wage increases via ballot initiatives with support from Republican elites and voters in each state. Similarly, since 2017 voters have circumvented Republican-controlled state legislatures to expand Medicaid through ballot measures in South Dakota, Maine, Missouri, Oklahoma, Idaho, Nebraska, and Utah [76] These examples underscore the viability of ballot measure campaigns as a mechanism for advancing workers’ rights in non-traditional political strongholds and highlight the potential for a reconfiguration of class consciousness among working voters.

We would be remiss not to mention the limitations of this study. First, although the study is adequately powered to detect medium-sized average treatment effects across the five experimental conditions, it is not necessarily powered to detect smaller effects, interaction effects, or heterogeneous treatment effects across multiple subgroups, which typically require substantially larger samples. Second, while respondents were randomly assigned to treatment conditions, randomization ensures balance only in expectation; in finite samples, residual imbalances on observed or unobserved covariates may remain. Third, although survey weights improve alignment between the sample and population benchmarks on key demographic and political characteristics, weighting does not fully eliminate the possibility of residual non-representativeness, particularly on unmeasured characteristics. In addition, the study relies on self-reported survey measures, which may be subject to measurement error or social desirability bias. Finally, there may be generalizability concerns related to the sample and the experimental design. The dependent variable captures short-term responses to the treatments and may not reflect longer-term attitudes or behaviors. Further, no matter how representative of the country, our puzzle centers on a single state, potentially limiting the extent to which the findings can be generalized beyond the study context.

In sum, this study not only contributes to our understanding of the intricate interplay between partisanship, union movements, and support for labor rights initiatives but also offers valuable insights into effective strategies for promoting workers’ rights in polarized political environments. Moving forward, further research is warranted to explore the enduring impact of these findings on the trajectory of labor advocacy and policy formulation, particularly in the context of a contentious American political climate and a potential changing patterns of partisan allegiance.

## Supporting information

S1 Appendix Supplemental tables. (DOCX)
