# Supplementary material for: Converting the “union curious”? Rights-based, pro-worker arguments and Republican support for expanding collective bargaining: The case of the Illinois Workers’ Rights Amendment
Source: PLoS One. 2026 Feb 6;21(2):e0335702. doi: 10.1371/journal.pone.0335702 (PMC12880653; doi:10.1371/journal.pone.0335702)
Supplement: S1 Appendix — (DOCX) [file pone.0335702.s001.docx]

**APPENDIX – SUPPLEMENTAL TABLES**

Table S1: Descriptions of Pre-Treatment Covariates

|  | Mean | Std. Dev | Minimum | Maximum | Type |
| --- | --- | --- | --- | --- | --- |
| Pro-WRA Argument | 0.203 | 0.402 | 0 | 1 | Binary |
| Anti-WRA Argument | 0.202 | 0.401 | 0 | 1 | Binary |
| Public Sector Endorsement | 0.202 | 0.402 | 0 | 1 | Binary |
| Private Sector Endorsement | 0.200 | 0.397 | 0 | 1 | Binary |
| Democrat | 0.540 | 0.499 | 0 | 1 | Binary |
| Republican | 0.410 | 0.492 | 0 | 1 | Binary |
| Independent | 0.050 | 0.218 | 0 | 1 | Binary |
| Union Household | 0.161 | 0.368 | 0 | 1 | Binary |
| Working Class | 0.352 | 0.478 | 0 | 1 | Binary |
| Ideology | 3.916 | 1.702 | 1 | 7 | Ordinal |
| Income | 3.210 | 1.538 | 1 | 6 | Ordinal |
| Age | 2.776 | 0.948 | 1 | 4 | Ordinal |
| Female | 0.540 | 0.499 | 0 | 1 | Binary |
| White | 0.745 | 0.436 | 0 | 1 | Binary |
| Unemployed | 0.149 | 0.356 | 0 | 1 | Binary |

- **Union Household-** Union membership has the potential to explain both Democratic Party membership and attitudes toward the WRA. Because of its potential as a confounding variable, we asked respondents, “Do you or anyone else in your household belong to a labor union or to an employee association similar to a union?” Respondents answered “Yes” or “No” to create a binary variable.
- **Working Class-** Working class membership is associated with party membership [63] and potentially with union attitudes. To measure this characteristic we asked the open-ended, “What kind of work do you do? For example, registered nurse, janitor, cashier, auto mechanic.” We took the free response answers and coded the variable according to the measure as defined by Carnes and Lupu [63]. Respondents are considered working class if their self-identified employment is in manual labor, the service industry jobs, clerical work, or a union job.
- **Ideology-**Respondent ideology is related to partisanship and possibly to WRA attitudes. We included the following question in our survey: “One way that people talk about politics in the United States is in terms of liberal, conservative, and moderate ideology. The political views people might hold are often arranged from extremely liberal (1) to extremely conservative (7). Using that scale, where do you place yourself?”
- **Income-** Respondent income has the potential to be correlated with partisan and union attitudes. We asked respondents a multiple-choice question “Thinking back over the last year, what was your approximate household income before taxes?” Possible answers were: “Less than $24,999 per year”, “$25,000—$49,999 per year”, “$50,000—$74,999 per year”, “$75,000—$99,999 per year”, “$100,000—$149,999 per year”, “$150,000 or more per year.” We then scaled this variable from 1 ($24,999 or less) to 6 ($150,000 or more).
- **Age-** Given the role of age as a factor in partisan politics and its potential to affect attituded towards unions we asked respondents a multiple-choice question “What is your age?” Options included: “18 to 29 years old”, “30 to 44 years old”, “45 to 64 years old”, or “65 years or older.” We then scaled this variable from 1 (18-29-year-olds) to 4 (65 or older).
- **Female-** We asked respondents, “What is your gender?” Respondents could respond: “Male, “Female”, or “Other/Non-Binary”. We transformed this response into a binary variable where female respondents are coded as 1 and all other respondents coded as 0.
- **White-** Race and ethnicity are historically related both to partisan attitudes and union membership. To account for this, we asked respondents “Now think of your own background in racial and ethnic terms. How would you describe your race and ethnicity? (Please, select all that apply)” Options included: “African American”, “White”, “Hispanic/Latino”. “Asian”, “Native American”, and “Other (Please specify).” We transformed this response into a binary variable consistent with existing literature where respondents that identified “White” as their sole race/ethnicity are coded as 1 and all other respondents coded as 0.
- **Unemployed**- Finally, employment status might serve as a predictor of both partisan membership and union support in unexpected ways. We asked respondents, “Which of the following best describes your current employment status? (Please, select all that apply.)” Options included: “Employed full-time”. “Employed part-time”, “Unemployed”, “Full-time student”, “Disabled and unable to work”, “Retired”, “At home parent.” These selections were transformed into a binary variable coded as 1 if a respondent indicated they were unemployed, retired, a stay-at-home parent, or unable to work due to a disability, and 0 otherwise.

Table S2: Logit Models of the Effect of Partisanship on WRA Support

|  | *Dependent variable:* | | | |
| --- | --- | --- | --- | --- |
|  |  | | | |
|  | Support for WRA | | | |
|  | (1) | (2) | (3) | (4) |
|  | | | | |
| Democrat | 0.895^*^ |  |  | 1.157^*^ |
|  | (0.179) |  |  | (0.332) |
|  |  |  |  |  |
| Republican |  | -0.666^*^ |  | 0.325 |
|  |  | (0.184) |  | (0.343) |
|  |  |  |  |  |
| Independent |  |  | -0.828^*^ |  |
|  |  |  | (0.326) |  |
|  |  |  |  |  |
| Union Household | 0.680^*^ | 0.659^*^ | 0.648^*^ | 0.681^*^ |
|  | (0.180) | (0.179) | (0.177) | (0.180) |
|  |  |  |  |  |
| Pro-WRA Argument | -0.036 | -0.043 | 0.013 | -0.027 |
|  | (0.227) | (0.226) | (0.223) | (0.227) |
|  |  |  |  |  |
| Anti-WRA Argument | -0.495^*^ | -0.518^*^ | -0.471^*^ | -0.486^*^ |
|  | (0.229) | (0.228) | (0.227) | (0.229) |
|  |  |  |  |  |
| Public Sector Endorsement | -0.234 | -0.222 | -0.203 | -0.234 |
|  | (0.226) | (0.225) | (0.223) | (0.226) |
|  |  |  |  |  |
| Private Sector Endorsement | -0.316 | -0.324 | -0.267 | -0.307 |
|  | (0.231) | (0.230) | (0.228) | (0.231) |
|  |  |  |  |  |
| Working Class | -0.112 | -0.098 | -0.074 | -0.111 |
|  | (0.158) | (0.157) | (0.156) | (0.158) |
| Union Household | -0.389^*^ | -0.428^*^ | -0.545^*^ | -0.399^*^ |
|  | (0.056) | (0.057) | (0.048) | (0.057) |
|  |  |  |  |  |
| Income | -0.056 | -0.054 | -0.061 | -0.057 |
|  | (0.051) | (0.051) | (0.050) | (0.051) |
|  |  |  |  |  |
| Age | -0.084 | -0.064 | -0.079 | -0.089 |
|  | (0.082) | (0.082) | (0.081) | (0.083) |
|  |  |  |  |  |
| Female | -0.183 | -0.168 | -0.152 | -0.182 |
|  | (0.147) | (0.146) | (0.145) | (0.147) |
|  |  |  |  |  |
| White | 0.090 | 0.027 | -0.084 | 0.085 |
|  | (0.180) | (0.178) | (0.175) | (0.180) |
|  |  |  |  |  |
| Unemployed | -0.060 | -0.078 | -0.124 | -0.064 |
|  | (0.216) | (0.215) | (0.213) | (0.216) |
|  |  |  |  |  |
| Constant | 1.516^*^ | 2.408^*^ | 2.751^*^ | 1.302^*^ |
|  | (0.441) | (0.385) | (0.380) | (0.497) |
|  |  |  |  |  |
|  | | | | |
| Observations | 1,000 | 1,000 | 1,000 | 1,000 |
|  | | | | |
| *Note:* all logit models | ^*^p<0.05 | | | |

Table S3: Logit Models of Effect of Arguments on WRA Attitudes

|  | *Dependent variable:* | | |
| --- | --- | --- | --- |
|  | Support for WRA | | |
|  | (1) | (2) | (3) |
|  | | | |
| Pro-Argument * Republican |  | 0.687^**^ |  |
|  |  | (0.344) |  |
| Pro-Argument * Democrat | -0.431 |  |  |
|  | (0.343) |  |  |
| Pro-Argument * Independent |  |  | -1.277 |
|  |  |  | (0.871) |
| Pro-WRA Argument | 0.151 | -0.383 | 0.071 |
|  | (0.286) | (0.255) | (0.209) |
| Anti-WRA Argument | -0.447^*^ | -0.501^*^ | -0.359^*^ |
|  | (0.224) | (0.223) | (0.207) |
| Public Sector Endorsement | -0.204 | -0.198 | -0.096 |
|  | (0.220) | (0.219) | (0.204) |
| Private Sector Endorsement | -0.274 | -0.294 | -0.089 |
|  | (0.223) | (0.222) | (0.207) |
| Democrat | 1.685^*^ |  |  |
|  | (0.166) |  |  |
| Republican |  | -1.627^*^ |  |
|  |  | (0.168) |  |
| Independent |  |  | -0.517 |
|  |  |  | (0.353) |
| Union Household | 0.683^*^ | 0.660^*^ | 0.565^*^ |
|  | (0.195) | (0.192) | (0.181) |
| Working Class | -0.232 | -0.228 | -0.243^*^ |
|  | (0.153) | (0.151) | (0.143) |
| Income |  |  |  |
|  | -0.073 | -0.067 | -0.075 |
| Age | (0.049) | (0.049) | (0.046) |
|  |  |  |  |
| Female | -0.176^*^ | -0.145^*^ | -0.208^*^ |
|  | (0.078) | (0.078) | (0.074) |
| White |  |  |  |
|  | -0.231 | -0.229 | -0.153 |
| Unemployed | (0.142) | (0.140) | (0.132) |
|  |  |  |  |
| Constant | -0.096 | 1.444^*^ | 1.229^*^ |
|  | (0.361) | (0.346) | (0.325) |
| Observations | 1,000 | 1,000 | 1,000 |
| *Note: all logit models* |  |  | ^*^p<0.05 |

Table S4: Logit Models of Effect of Endorsements on WRA Attitudes

|  | *Dependent variable:* | |
| --- | --- | --- |
|  | Support for WRA | |
|  | (1) | (2) |
|  | | |
| Republican | -0.589^*^ |  |
|  | (0.194) |  |
|  |  |  |
| Private Sector Endorsement | -0.159 | -0.296 |
|  | (0.266) | (0.229) |
|  |  |  |
| Democrat |  | 0.775^*^ |
|  |  | (0.193) |
|  |  |  |
| Public Sector Endorsement | -0.214 | -0.583^*^ |
|  | (0.224) | (0.317) |
|  |  |  |
| Union Household | 0.659^*^ | 0.684^*^ |
|  | (0.179) | (0.180) |
| Pro-WRA Argument | -0.035 | -0.026 |
|  | (0.225) | (0.225) |
| Anti-WRA Argument | -0.505^*^ | -0.483^*^ |
|  | (0.227) | (0.227) |
| Working Class | -0.100 | -0.127 |
|  | (0.157) | (0.159) |
| Ideology | -0.425^*^ | -0.388^*^ |
|  | (0.057) | (0.056) |
| Income | -0.054 | -0.058 |
|  | (0.051) | (0.051) |
|  |  |  |
| Age | -0.062 | -0.091 |
|  | (0.082) | (0.083) |
|  |  |  |
| Female | -0.174 | -0.196 |
|  | (0.146) | (0.148) |
|  |  |  |
| White | 0.035 | 0.089 |
|  | (0.178) | (0.180) |
|  |  |  |
| Unemployed | -0.071 | -0.080 |
|  | (0.215) | (0.216) |
|  |  |  |
| Republican * Private Sector Endorsement | -0.506 |  |
|  | (0.407) |  |
|  |  |  |
| Democrat * Public Sector Endorsement |  | 0.614^*^ |
|  |  | (0.377) |
|  |  |  |
| Constant | 2.351^*^ | 1.611^*^ |
|  | (0.387) | (0.445) |
|  |  |  |
|  | | |
| Observations | 1,000 | 1,000 |
|  | | |
| *Note: all logit models* | ^*^p<0.05 | |

Table S5: Aggregate Support for WRA By Partisanship

|  | | | |
| --- | --- | --- | --- |
|  | *Dependent variable:* | | |
|  |  | | |
|  | Support for WRA | | |
|  | (1) | (2) | (3) |
|  | | | |
| Democrat | 1.579^*^ |  |  |
|  | (0.138) |  |  |
|  |  |  |  |
| Republican |  | -1.478^*^ |  |
|  |  | (0.139) |  |
|  |  |  |  |
| Independent |  |  | -0.721^*^ |
|  |  |  | (0.309) |
|  |  |  |  |
| Constant | -0.931^*^ | 0.523^*^ | -0.028 |
|  | (0.104) | (0.085) | (0.065) |
|  |  |  |  |
|  | | | |
| Observations | 1,000 | 1,000 | 1,000 |
|  | | | |
| *Note: all bivariate logit models* | ^*^p<0.05 | | |

Table S6: Aggregate Support for WRA By Treatment Group

|  | | | | | | | |
| --- | --- | --- | --- | --- | --- | --- | --- |
|  | *Dependent variable:* | | | | | | |
|  |  | | | | | | |
|  | Support for WRA | | | | | | |
|  | (1) | | (2) | (3) | | | (4) |
|  | | | | | | | |
| Pro-WRA Argument | 0.042 | |  |  | | |  |
|  | (0.201) | |  |  | | |  |
| Anti-WRA Argument |  | | -0.268 |  | | |  |
|  |  | | (0.201) |  | | |  |
| Public Sector Endorsement |  | |  | -0.166 | | |  |
|  |  | |  | (0.201) | | |  |
| Private Sector Endorsement |  | |  |  | | | -0.102 |
|  |  | |  |  | | | (0.202) |
| Constant | 0.037 | | 0.037 | 0.037 | | | 0.369 |
|  | (0.142) | | (0.143) | (0.142) | | | (0.143) |
|  | | | | | | | |
| Observations | 400 | 399 | | | 399 | 393 | |
|  | | | | | | | |
| *Note: all bivariate logit models* | ^*^p<0.05 | | | | | | |

Table S7: Democrats Support for WRA Relative to Other Partisan Groups

|  | | | |
| --- | --- | --- | --- |
|  | *Dependent variable:* | | |
|  |  | | |
|  | Support for WRA | | |
|  | (1) | (2) | (3) |
|  | *Democrats vs. All Others* | *Democrats vs. Republicans* | *Democrats vs. Independents* |
|  | | | |
| Democrat | 0.895^*^ | 0.819^*^ | 1.129^*^ |
|  | (0.179) | (0.191) | (0.340) |
|  |  |  |  |
| Union Household | 0.680^*^ | 0.697^*^ | 1.002^*^ |
|  | (0.180) | (0.186) | (0.253) |
|  |  |  |  |
| Pro-WRA Argument | -0.036 | 0.017 | -0.216 |
|  | (0.227) | (0.234) | (0.302) |
|  |  |  |  |
| Anti-WRA Argument | -0.495^*^ | -0.535^*^ | -0.610^*^ |
|  | (0.229) | (0.237) | (0.303) |
|  |  |  |  |
| Public Sector Endorsement | -0.234 | -0.218 | -0.070 |
|  | (0.226) | (0.231) | (0.307) |
|  |  |  |  |
| Private Sector Endorsement | -0.316 | -0.379 | -0.166 |
|  | (0.231) | (0.238) | (0.303) |
|  |  |  |  |
| Working Class | -0.112 | -0.105 | -0.177 |
|  | (0.158) | (0.162) | (0.204) |
|  |  |  |  |
| Ideology | -0.389^*^ | -0.404^*^ | -0.407^*^ |
|  | (0.056) | (0.058) | (0.076) |
|  |  |  |  |
| Income | -0.056 | -0.035 | -0.114^*^ |
|  | (0.051) | (0.053) | (0.066) |
|  |  |  |  |
| Age | -0.084 | -0.085 | 0.041 |
|  | (0.082) | (0.085) | (0.104) |
|  |  |  |  |
| Female | -0.183 | -0.147 | -0.303 |
|  | (0.147) | (0.151) | (0.191) |
|  |  |  |  |
| White | 0.090 | 0.018 | -0.025 |
|  | (0.180) | (0.186) | (0.211) |
|  |  |  |  |
| Unemployed | -0.060 | -0.014 | -0.167 |
|  | (0.216) | (0.223) | (0.278) |
|  |  |  |  |
| Constant | 1.516^*^ | 1.598^*^ | 1.309^*^ |
|  | (0.441) | (0.470) | (0.594) |
|  |  |  |  |
|  | | | |
| Observations | 1,000 | 950 | 590 |
|  | | | |
| *Note:* all logit models | ^*^p<0.05 | | |

Table S8: Republicans Support for WRA Relative to Other Partisan Groups

|  | | | |
| --- | --- | --- | --- |
|  | *Dependent variable:* | | |
|  |  | | |
|  | Support for WRA | | |
|  | (1) | (2) | (3) |
|  | *Republicans vs. All Others* | *Republicans vs. Democrats* | *Republicans vs. Independents* |
|  | | | |
| Republican | -0.666^*^ | -0.819^*^ | 0.375 |
|  | (0.184) | (0.191) | (0.361) |
|  |  |  |  |
| Union Household | 0.659^*^ | 0.697^*^ | 0.339 |
|  | (0.179) | (0.186) | (0.269) |
|  |  |  |  |
| Pro-WRA Argument | -0.043 | 0.017 | 0.110 |
|  | (0.226) | (0.234) | (0.330) |
|  |  |  |  |
| Anti-WRA Argument | -0.518^*^ | -0.535^*^ | -0.289 |
|  | (0.228) | (0.237) | (0.335) |
|  |  |  |  |
| Public Sector Endorsement | -0.222 | -0.218 | -0.557 |
|  | (0.225) | (0.231) | (0.349) |
|  |  |  |  |
| Private Sector Endorsement | -0.324 | -0.379 | -0.404 |
|  | (0.230) | (0.238) | (0.360) |
|  |  |  |  |
| Working Class | -0.098 | -0.105 | -0.096 |
|  | (0.157) | (0.162) | (0.250) |
|  |  |  |  |
| Ideology | -0.428^*^ | -0.404^*^ | -0.386^*^ |
|  | (0.057) | (0.058) | (0.091) |
|  |  |  |  |
| Income | -0.054 | -0.035 | -0.020 |
|  | (0.051) | (0.053) | (0.080) |
|  |  |  |  |
| Age | -0.064 | -0.085 | -0.301^*^ |
|  | (0.082) | (0.085) | (0.133) |
|  |  |  |  |
| Female | -0.168 | -0.147 | -0.169 |
|  | (0.146) | (0.151) | (0.231) |
|  |  |  |  |
| White | 0.027 | 0.018 | 0.499 |
|  | (0.178) | (0.186) | (0.363) |
|  |  |  |  |
| Unemployed | -0.078 | -0.014 | -0.092 |
|  | (0.215) | (0.223) | (0.334) |
|  |  |  |  |
| Constant | 2.408^*^ | 2.417^*^ | 1.391^*^ |
|  | (0.385) | (0.399) | (0.682) |
|  |  |  |  |
|  | | | |
| Observations | 1,000 | 950 | 460 |
|  | | | |
| *Note:* all logit models | ^*^p<0.05 | | |

Table S9: Independents Support for WRA Relative to Other Partisan Groups

|  | | | |
| --- | --- | --- | --- |
|  | *Dependent variable:* | | |
|  |  | | |
|  | Support for WRA | | |
|  | (1) | (2) | (3) |
|  | *Independents vs. All Others* | *Independents vs. Democrats* | *Independents vs. Republicans* |
|  | | | |
| Independent | -0.828^*^ | -1.129^*^ | -0.375 |
|  | (0.326) | (0.340) | (0.361) |
|  |  |  |  |
| Union Household | 0.648^*^ | 1.002^*^ | 0.339 |
|  | (0.177) | (0.253) | (0.269) |
|  |  |  |  |
| Pro-WRA Argument | 0.013 | -0.216 | 0.110 |
|  | (0.223) | (0.302) | (0.330) |
|  |  |  |  |
| Anti-WRA Argument | -0.471^*^ | -0.610^*^ | -0.289 |
|  | (0.227) | (0.303) | (0.335) |
|  |  |  |  |
| Public Sector Endorsement | -0.203 | -0.070 | -0.557 |
|  | (0.223) | (0.307) | (0.349) |
|  |  |  |  |
| Private Sector Endorsement | -0.267 | -0.166 | -0.404 |
|  | (0.228) | (0.303) | (0.360) |
|  |  |  |  |
| Working Class | -0.074 | -0.177 | -0.096 |
|  | (0.156) | (0.204) | (0.250) |
|  |  |  |  |
| Ideology | -0.545^*^ | -0.407^*^ | -0.386^*^ |
|  | (0.048) | (0.076) | (0.091) |
|  |  |  |  |
| Income | -0.061 | -0.114^*^ | -0.020 |
|  | (0.050) | (0.066) | (0.080) |
|  |  |  |  |
| Age | -0.079 | 0.041 | -0.301^*^ |
|  | (0.081) | (0.104) | (0.133) |
|  |  |  |  |
| Female | -0.152 | -0.303 | -0.169 |
|  | (0.145) | (0.191) | (0.231) |
|  |  |  |  |
| White | -0.084 | -0.025 | 0.499 |
|  | (0.175) | (0.211) | (0.363) |
|  |  |  |  |
| Unemployed | -0.124 | -0.167 | -0.092 |
|  | (0.213) | (0.278) | (0.334) |
|  |  |  |  |
| Constant | 2.751^*^ | 2.438^*^ | 1.766^*^ |
|  | (0.380) | (0.495) | (0.698) |
|  |  |  |  |
|  | | | |
| Observations | 1,000 | 590 | 460 |
|  | | | |
| *Note:* all logit models | ^*^p<0.05 | | |
